# Nicotine Exerts Cytotoxic Effects in a Panel of Healthy Cell Lines and Strong Irritating Potential on Blood Vessels

**DOI:** 10.3390/ijerph19148881

**Published:** 2022-07-21

**Authors:** Doina Chioran, Adrian Sitaru, Ioana Macasoi, Iulia Pinzaru, Cristian Andrei Sarau, Cristina Dehelean, Stefania Dinu, Camelia Szuhanek, Irina Nicoleta Zetu, Andra Cristine Serafin, Mircea Rivis, Marioara Poenaru, Razvan Dragoi

**Affiliations:** 1 Faculty of Dental Medicine, “Victor Babes” University of Medicine and Pharmacy Timisoara, Eftimie Murgu Square No. 2, 300041 Timisoara, Romania; chioran.doina@umft.ro (D.C.); dinu.stefania@umft.ro (S.D.); cameliaszuhanek@umft.ro (C.S.); andrada_serafin@yahoo.ro (A.C.S.); rivis.mircea@umft.ro (M.R.); 2 Faculty of Medicine, “Victor Babes” University of Medicine and Pharmacy Timisoara, Eftimie Murgu Square No. 2, 300041 Timisoara, Romania; adrian.sitaru@umft.ro (A.S.); marioara.poenaru@gmail.com (M.P.); dragoi.razvan@umft.ro (R.D.); 3 Departament of Toxicology and Drug Industry, Faculty of Pharmacy, “Victor Babes” University of Medicine and Pharmacy Timisoara, Eftimie Murgu Square No. 2, 300041 Timisoara, Romania; macasoi.ioana@umft.ro (I.M.); cadehelean@umft.ro (C.D.); 4 Research Center for Pharmaco-Toxicological Evaluations, Faculty of Pharmacy, “Victor Babes” University of Medicine and Pharmacy Timisoara, Eftimie Murgu Square No. 2, 300041 Timisoara, Romania; 5 Faculty of Dental Medicine, University of Medicine and Pharmacy “Grigore T. Popa” Iasi, University Street No. 16, 700115 Iasi, Romania; nicoleta.zetu@gmail.com

**Keywords:** nicotine, electronic cigarettes, HET-CAM assay, in vitro toxicity, cytotoxicity

## Abstract

The use of tobacco products is a major global public health issue, as it is the leading cause of preventable death worldwide. In addition, nicotine (NIC) is a key component of electronic and conventional cigarettes. Although nicotine’s addictive potential is well known, its health effects are not entirely understood. Thus, the main objective of the present study was to evaluate its toxicological profile both in vitro, at the level of three healthy cell lines, and in ovo, at the level of the chorioallantoic membrane. Five different concentrations of nicotine were used in keratinocytes, cardiomyocytes, and hepatocytes for the purpose of evaluating cell viability, cell morphology, and its impact on nuclei. Additionally, the hen’s egg test on the chorioallantoic membrane (HET-CAM) method was used to assess the biocompatibility and irritant potential of the chorioallantoic membrane. Across all cell lines studied, nicotine was proven to be significantly damaging to cell viability, with the highest concentration tested resulting in less than 2% viable cells. Moreover, the morphology of cells changed dramatically, with alterations in their shape and confluence. Nicotine-induced cell death appears to be apoptotic, based on its impact on the nucleus. In addition, nicotine was also found to have a very strong irritating effect on the chorioallantoic membrane. In conclusion, nicotine has an extremely strong toxicological profile, as demonstrated by the drastic reduction of cell viability and the induction of morphological changes and nuclear alterations associated with cellular apoptosis. Additionally, the HET-CAM method led to the observation of a strong irritating effect associated with nicotine.

## 1. Introduction

Tobacco use is a public health concern, especially in countries with low or middle incomes [1,2]. A modern problem associated with tobacco is the introduction of electronic cigarettes, the nicotine content of which is often mislabeled [3,4,5,6].

Nicotine is a dibasic compound, of which the pharmacokinetic properties are strongly affected by the pH of the solution being absorbed through the oral mucosa, lungs, skin, and intestines; however, the concentration of lipophilic nicotine capable of entering all biological membranes increases as the pH of the solution increases [7]. After absorption, nicotine undergoes hepatic metabolism, performed in two phases, with the formation of metabolites such as N-nitrosonornicotine (NNN) and 4-(methylnitrosamino)-1-(3-pyridyl)-1-butanone (NNK), which are known for their carcinogenic potential [8].

Nicotine is a highly addictive psychoactive substance. In addition, it has a high toxicological profile, belonging to the category of the most harmful substances. However, in terms of the effect of NIC on smokers, it is more difficult to establish a direct causal relationship between it and systemic toxic effects, due to the fact that both regular cigarettes and e-cigarettes contain a multitude of other substances with toxic potential [9]. Several tobacco-related diseases have become more prevalent in recent years as a result of the rise in tobacco consumption. There are many known consequences of smoking in adults, including the appearance of cancerous processes in the organs exposed to tobacco, as well as several chronic conditions such as eye disorders and cardiovascular, lung, and periodontal diseases [2].

As far as nicotine’s effects on the skin are concerned, they are generally well known. Therefore, nicotine interferes with wound healing by deteriorating the epithelial layer and altering the rheological properties of the blood, inducing vasoconstriction. The alteration of collagen metabolism contributes to the slow process of wound healing and this can also contribute to the aging process [10]. NIC has been linked to premature skin aging and delayed wound healing as a result of nicotine receptors located in keratinocytes of the skin [11].

Regarding its effect on the liver, smoking and nicotine undoubtedly cause adverse effects through three main mechanisms, namely, (i) direct and indirect toxicity, (ii) immunological toxicity, and (iii) oncogenic toxicity. Tobacco’s direct toxicity includes increased oxidative stress, as well as an increase in the presence of proinflammatory cytokines. Indirect toxicity occurs due to the production of carboxyhemoglobin, which has a detrimental impact on oxygen transport capacity, which leads to the excess availability of catabolic iron in the hepatocytes, causing oxidative stress [12]. The oncological effects associated with smoking are due to the contents of potentially carcinogenic substances, such as hydrocarbons, nitrosamine, and vinyl chloride [13]. Nicotine plays a significant role in the immune mechanisms that cause liver damage. In this manner, nicotine inhibits lymphocyte proliferation and differentiation, thereby impairing antibody synthesis [14].

In the cardiovascular system, smoking exerts toxic effects in a variety of ways, including the induction of oxidative stress, chronic inflammation, hemodynamic stress, arrhythmias, impaired endothelial function and oxygen transport to erythrocytes, increased insulin resistance, and the development of diabetes [15]. As a result of increased cardiac contractility and systemic vasoconstriction, nicotine promotes an increase in the heart rate [16]. Nicotine also decreases cell viability in cardiomyocytes by triggering apoptosis due to increased oxidative stress [17].

Taking these factors into account, the present study was designed to determine, in vitro, the effects of nicotine on the viability, morphology, and structure of the nucleus on three healthy cell lines, namely, keratinocytes—HaCaT, hepatocytes—HepaRG, and cardiomyocytes—H9C2 (2-1). In addition, the potential irritant effect was assessed by means of the hen’s egg test on the chorioallantoic membrane.

## 2. Materials and Methods

### 2.1. Reagents

Reagents used for in vitro experiments: trypsin-EDTA solution, phosphate saline buffer (PBS), dimethyl sulfoxide (DMSO), fetal calf serum (FCS), penicillin/streptomycin, insulin from bovine pancreas, hydrocortisone 21-hemisuccinate sodium, and MTT (3-(4,5-dimethylthiazol-2-yl)-2,5-diphenyltetrazolium bromide) reagents were purchased from Sigma-Aldrich, Merck KgaA (Darmstadt, Germany). The cell media used, William’s E Medium (Gibco™; 12551032), was purchased from Gibco, Waltham, MA, USA, and Dulbecco’s modified Eagle’s medium (DMEM; 30–2002™) was purchased from the ATCC (American Type Cell Collection, Manassas, VA, USA). All reagents were analytically pure and proper for cell culture use. Nicotine ((−)-1-Methyl-2-(3-pyridyl) pyrrolidine; purity ≥ 99%) was acquired from Sigma-Aldrich, Merck KgaA (Darmstadt, Germany).

### 2.2. Cell Culture

In vitro experiments were carried out with HaCaT keratinocytes from CLS Cell Lines Service GmbH (Eppelheim, Germany); HepaRG hepatocytes supplied by Gibco, Ltd. (catalog number HPRGC10); and H9C2(2-1) cardiomyocytes provided by ATCC (American Type Cell Collection, Manassas, VA, USA). DMEM (containing 10% FBS and 1% penicillin (100 U/mL)-streptomycin (100 g/mL) was used to culture HaCaT and H9C2(2-1) cells. Specifically, HepaRG cells were cultured in defined media, represented by William’s E Medium containing 4 g/mL insulin from bovine pancreatic tissue, 5 μM hydrocortisone 21-hemisuccinate sodium, 10% FCS, and 1% penicillin (100 U/mL)-streptomycin (100 g/mL) mixture. A standard temperature (37 °C) and 5% CO_2_ were maintained throughout the experiment.

### 2.3. Cellular Viability Assessment

The viability of the cells was assessed using the MTT method. Therefore, the following steps were completed: (i) the cells were cultured in 96-well plates at a density of 1 × 10^4^ cells per well; (ii) the cells were stimulated with five concentrations of nicotine (0.1, 10, 25, 50, and 100 μL/mL—prepared in the specific culture medium for each cell line) at a time interval of 24 h (after reaching a suitable confluence of approximately 90%); (iii) after 24 h, the stimulation medium was replaced with 100 μL/well of fresh culture medium and 10 μL/well of MTT reagent, and the plates were subsequently incubated at 37 °C for 3 h; (iv) 100 μL/well of the solubilization solution was added, and the plates were kept at room temperature for 30 min, protected from light; and (v) the absorbances were measured at two wavelengths of 570 nm and 630 nm using a Cytation 5 system (BioTek Instruments Inc., Winooski, VT, USA).

### 2.4. Cellular Morphology

To further comprehend the effects of nicotine induced in the selected cells in the present study, the morphology of the cells was evaluated after 24 h of stimulation. A microscopic evaluation was carried out by photographing the cells under bright-field illumination. The pictures were analyzed using Gen5™ Microplate Data Collection and Analysis Software (BioTek Instruments Inc., Winooski, VT, USA).

### 2.5. Nuclear Staining

A staining test with Hoechst 33342 was used to define the type of cell death induced by nicotine in the selected cell lines. In brief, the following steps were completed: (i) the cells were cultured in 12-well plates in 1 × 10^5^ cells/well number; (ii) the cells were stimulated with three concentrations of NIC (0.1, 10, and 25 μL/mL), after reaching a confluence of 80–90%; (iii) 24 h after this, the cell medium was removed and a volume of 500 μL/well of 1:2000 staining solution diluted in PBS was added; (iv) the plates were placed at room temperature for 10 min in the dark; and (v) the staining solution was washed three times with PBS. For data analysis, 5 μM staurosporin was used as a positive control for the induction of apoptosis, followed by incubation at 37 °C for 3 h. In order to calculate the apoptosis index, the formula described above was employed [18]:

$$\mathrm{AI}\;(\%)=\frac{Number\;of\;apoptotic\;cells}{Total\;number\;of\;cells}\times 100$$

### 2.6. Chorioallantoic Membrane (CAM) Assay

In this study, we examined the effect of nicotine on the vascular plexus of the chorioallatoic membrane using chicken eggs (*Gallus gallus domesticus*). Specifically, the preparation process was as follows: (i) the chicken eggs were washed and disinfected with 70% alcohol (*V*/*V*), after which they were incubated under normal temperatures and humidity; (ii) on the fourth day of incubation, a small hole was drilled in the egg’s shell, through which albumen was extracted in a volume of approximately 7–8 mL—this operation allowed the chorioallantoic membrane to detach from the inner shell of the hen’s egg, which affords easy visualization of the blood vessels; (iii) on the fifth day of incubation, a window was cut in the upper level of the hen’s egg, which was then covered with adhesive tape, and the eggs were then placed in the incubator until the experiment began.

### 2.7. Hen’s Egg Test on the Chorioallantoic Membrane (HET-CAM) Assay

To assess the biocompatibility and potential irritant effects of nicotine in the blood vessels, the HET-CAM method was employed. The experiment was performed on the ninth day following the incubation period. Nicotine was tested in three different concentrations, the lowest concentration previously tested in the cell viability test (0.1 μL/mL) and the highest concentrations previously tested in the cell viability test (50 and 100 μL/mL). Furthermore, for a better understanding of the effect induced by nicotine, two controls were employed: a negative one represented by distilled water, and a positive one represented by sodium dodecylsulfate (SDS), 1%. In order to attain complete coverage of the membrane surface, samples, along with the negative and positive controls, were applied in a volume of 600 μL to the chorioallantoic membrane. For five minutes, the vascular effects of these compounds were tracked, including lysis (*L*), coagulation (*C*), and hemorrhage (*H*). A stereomicroscope was used to observe the vascular effects (Discovery 8 Stereomicroscope from Zeiss, Göttingen, Germany) and images were taken with an Axio CAM 105 color camera from Zeiss 5 min before and after application of the samples. To quantify the irritant effect at the vascular level, the analytical method of calculating the irritation score (IS) was applied using the formula described above [19]:

$$IS=5\times \frac{301-H}{300}+7\times \frac{301-L}{300}+9\times \frac{301-C}{300}$$

The time of onset of hemorrhage was noted with *H*, vascular lysis with *L*, and intravascular coagulation with *C*. Irritation scoring is an easy method of classifying the irritant potential of a substance. Depending on the IS value, substances can be classified into three classes: (i) non-irritating (IS = 0–0.9); (ii) irritating (IS = 1–8.9), and (iii) severely irritating (IS = 9–21).

### 2.8. Statistical Analysis

Data are expressed as ±standard deviation (SD). Comparisons of differences between groups were performed using the one-way ANOVA test, followed by Dunett’s multiple post-test comparisons. The software used was GraphPad Prism version 9.3.1 for Windows (GraphPad Soft-ware, San Diego, CA, USA, www.graphpad.com). Statistically significant differences between data were labeled with * (* *p* < 0.1; ** *p* < 0.01; *** *p* < 0.001; **** *p* < 0.0001).

## 3. Results

### 3.1. Cellular Viability Assessment

**Figure 1:**
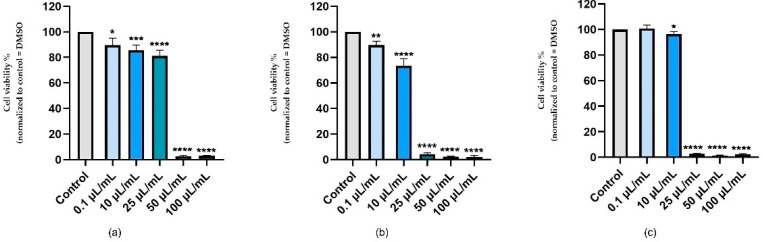
In vitro assessment of the effect of nicotine (0.1, 10, 25, 50, and 100 μL/mL) on the viability of: (**a**) keratinocytes (HaCaT), (**b**) hepatocytes (HepaRG), and (**c**) cardiomyocytes (H9C2(2-1)) after 24 h of treatment. The data are expressed as viability percentages (%) normalized to control cells (untreated cells) and expressed as mean values ± SD of three independent experiments performed in triplicate. To identify the statistical differences between the control and the nicotine-treated group, a one-way ANOVA was conducted, followed by Dunett’s multiple comparisons post-test (* *p* < 0.1; ** *p* < 0.01; *** *p* < 0.001; **** *p* < 0.0001).

As a means of assessing the effects of nicotine in vitro, three healthy cell lines were selected—keratinocytes, hepatocytes, and cardiomyocytes—and stimulated with five concentrations of nicotine (0.1, 10, 25, 50, and 100 μL/mL) for 24 h. In terms of the effect of nicotine on human keratinocytes, a slight reduction in cell viability was observed at the lowest concentrations tested (0.1–25 μL/mL) in a concentration-dependent manner. As a result of exposure to high concentrations (50 and 100 μL/mL), viability significantly decreased by about 3% for both concentrations (Figure 1a). Specifically, nicotine had a pronounced cytotoxic effect on hepatocytes, decreasing the viability of the cells in a concentration-dependent manner. It has been shown that even at the concentration of 25 μL/mL, a viability value of approximately 4% was recorded, though in the case of this concentration, there was no obvious cytotoxicity, as measured in the case of human keratinocytes. With the concentrations of 50 and 100 μL/mL, a similar trend was observed, with the value of viability being about 2.5% (Figure 1b). The viability of cardiomyocytes was also reduced in response to nicotine stimulation, in a similar manner to the decrease in viability reported for HepaRG cells. Consequently, in the first concentration test, no significant decline in the percentage of viable cells was observed, and in the case of the 10 μL/mL concentration test, the specific cell viability dropped slightly to approximately 96%. Additionally, the highest concentrations used in the present case resulted in a deterioration in cell viability, the value of which was around 2% (Figure 1c).

### 3.2. Cellular Morphology

**Figure 2:**
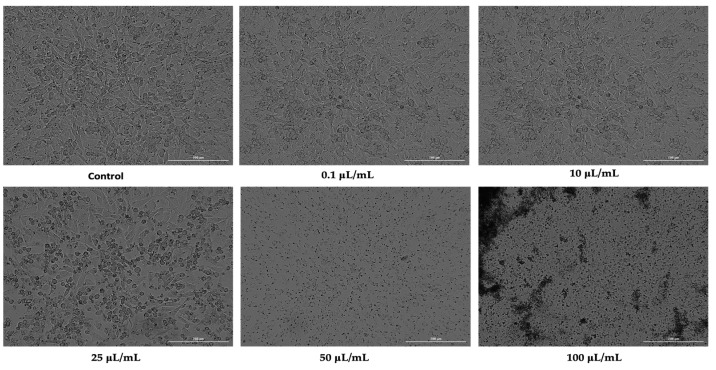
Morphology and confluence of keratinocytes (HaCaT) following 24 h treatment with nicotine at 0.1, 10, 25, 50, and 100 μL/mL. The scale bars indicate 200 µm.

For an overview of the effect of nicotine induced on the cell lines studied, cell morphology was assessed after 24 h of stimulation. As such, in the case of HaCaT cells, the first three concentrations tested (0.1, 10, and 25 μL/mL) did not cause drastic changes in terms of cell morphology or number. This concentration resulted in a slight rounding of the cells and a few detached cells. In contrast, at concentrations of 50 and 100 μL/mL, there were huge changes in the morphology of the cells, with a drastic drop in the number of cells attached to the plate, along with many rounded cells (Figure 2).

**Figure 3:**
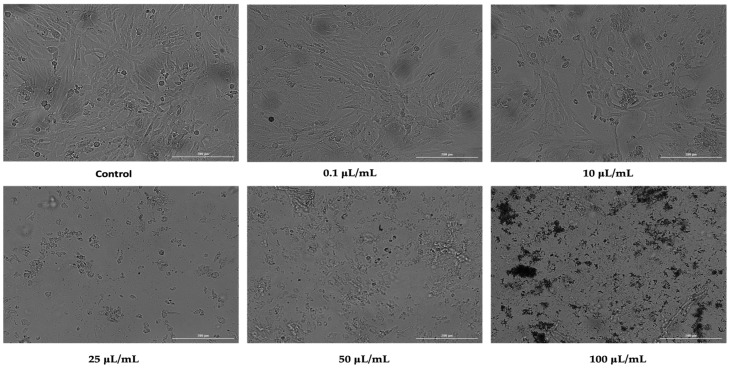
Morphology and confluence of hepatocytes (HepaRG) following 24 h treatment with nicotine at 0.1, 10, 25, 50, and 100 μL/mL. The scale bars indicate 200 µm.

Despite small differences, nicotine had a similar effect on HepaRG cells as it did on HaCaT cells. Thus, at concentrations of 0.1 and 10 μL/mL, it did not significantly affect the morphology of the cells, but it did reduce the confluence. Starting at a concentration of 25 μL/mL, the cell morphology underwent dramatic changes, with the cells becoming round and losing their attachment to the plaque, and a decrease in cell confluence was observed (Figure 3).

**Figure 4:**
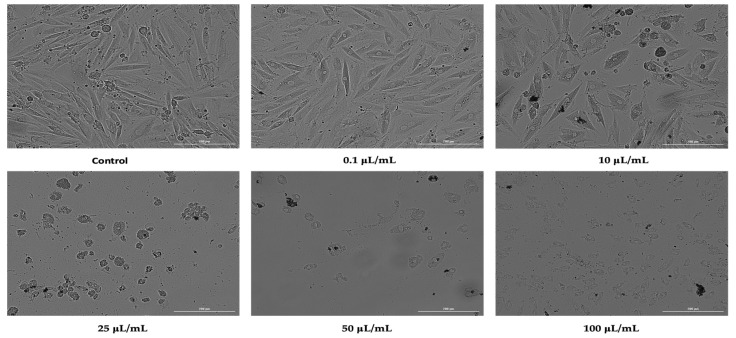
Morphology and confluence of cardiomyocytes (H9C2(2-1)) cells following 24 h treatment with nicotine 0.1, 10, 25, 50, and 100 μL/mL. The scale bars indicate 200 µm.

In H9C2 (2-1) cells, nicotine had a similar effect on morphology to that observed in HepaRG cells. Accordingly, the first two concentrations tested produced a slight morphological change and a reduction in the cell confluence of cardiomyocytes. Strong effects of reducing cell confluence and massive changes in cell morphology were recorded at concentrations of 25, 50, and 100 μL/mL, with the most intense effects being observed at the concentration of 100 μL/mL (Figure 4).

The effects observed in cell morphology were correlated with the results previously obtained in testing the influence of nicotine on cell viability.

### 3.3. Nuclear Staining

Having analyzed cell viability and morphology, the next step was to evaluate nicotine’s effects on the nuclei of the cells. Based on the fact that the highest tested concentrations (50 and 100 μL/mL) caused a massive reduction in cell viability, of around 2%, nuclear staining was observed only for the lowest concentrations (0.1, 10, and 25 μL/mL).

**Figure 5:**
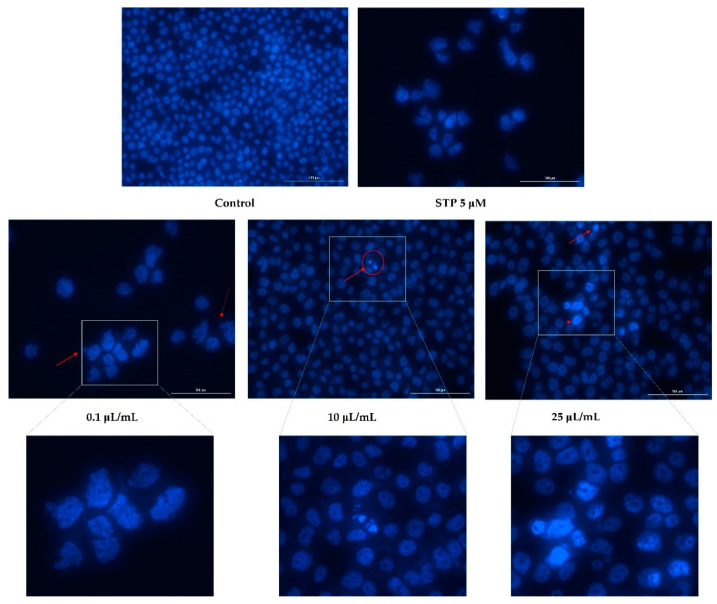
Keratinocytes—HaCaT nuclei stained with Hoechst 33342 dye after 24 h treatment with nicotine (0.1, 10, and 25 μL/mL). Staurosporine (5 µM) was used as the positive control for apoptotic changes at the nuclear level. The red arrows indicate signs of apoptosis. The scale bars represent 100 µm.

Figure 5 shows an apoptotic reaction occurring in the nuclei of HaCaT cells. In the case of the lowest concentration tested (0.1 μL/mL), apoptotic bodies and cell shrinkage were observed. In addition to these changes, at concentrations of 10 and 25 μL/mL, strong chromatin condensation and nuclear fragmentation were also observed (Figure 5).

**Figure 6:**
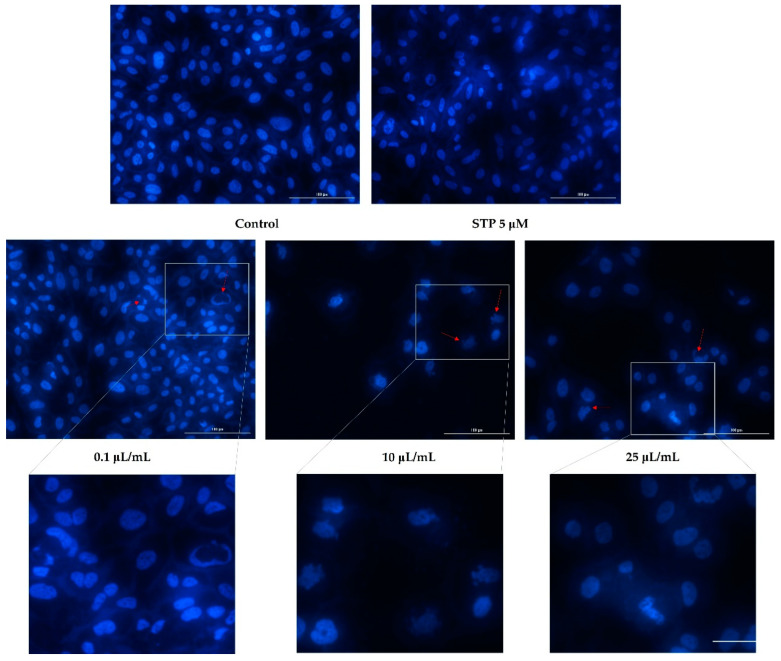
Hepatocytes—HepaRG nuclei stained with Hoechst 33342 dye after 24 h treatment with nicotine (0.1, 10, and 25 μL/mL). Staurosporine (5 µM) was used as the positive control for apoptotic changes at the nuclear level. The red arrows indicate signs of apoptosis. The scale bars represent 100 µm.

There was a decrease in the number of nuclei found in hepatocytes based on the concentration tested and as indicated by the previously obtained values of cell viability. Furthermore, Figure 6 shows that nicotine induced apoptotic changes in the nucleus. Consequently, the concentration of 0.1 μL/mL can lead to the condensation of chromatin, as well as shrinking of the nucleus. In addition to chromatin condensation, apoptotic bodies and nuclear fragmentation could be observed at concentrations of 10 and 25 μL/mL.

**Figure 7:**
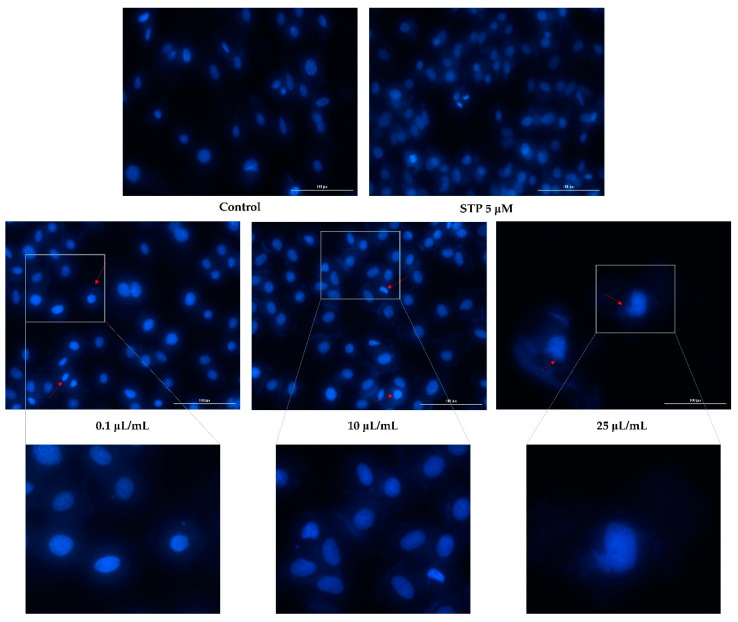
Cardiomyocytes—H9C2(2-1) nuclei stained with Hoechst 33342 dye after 24 h treatment with nicotine (0.1, 10, and 25 μL/mL). Staurosporine (5 µM) was used as the positive control for apoptotic changes at the nuclear level. The red arrows indicate signs of apoptosis. The scale bars represent 100 µm.

Additionally, nicotine had an apoptotic effect on cardiomyocytes as well. At the concentration of 0.1 μL/mL, a slight condensation of chromatin was detected, whereas in concentrations of 10 and 25 μL/mL, the formation of apoptotic bodies was observed, as well as strong condensation of chromatin (Figure 7).

**Figure 8:**
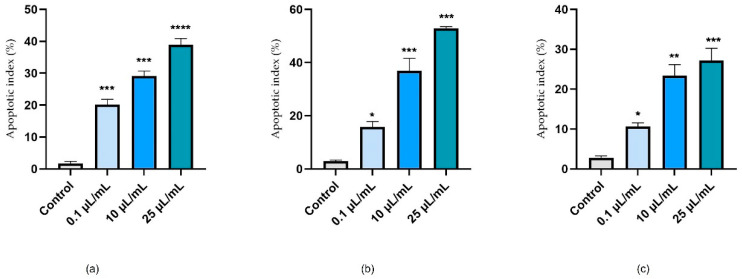
An analysis of the apoptotic index (AI) of HaCaT (**a**); HepaRG (**b**), and H9C2(2-1) (**c**) following 24 h of nicotine treatment (0.1, 10, and 25 μL/mL). Data are presented as an apoptotic index (%) normalized to controls and expressed as mean values ± SD of three independent experiments. The statistical differences between sontrols and the treated group were verified by applying a one-way ANOVA, followed by Dunnett’s multiple comparisons post-test (* *p* < 0.1; ** *p* < 0.01; *** *p* < 0.001; **** *p* < 0.0001).

Based on the calculated apoptosis index, nicotine appeared to have a pro-apoptotic effect, depending on the concentration tested, in all cell lines investigated. It can be seen that the apoptosis index increased, in accordance with the values obtained from the viability test. As a result, a concentration of 25 μL/mL strongly induced the apoptotic process in cells (Figure 8).

### 3.4. HET-CAM Assay

To assess the effects of nicotine in terms of irritation potential, the HET-CAM method was applied. In this study, nicotine was tested at three different concentrations, 0.1 μL/mL (the lowest concentration used to evaluate cellular viability), 50, and 100 μL/mL (the highest concentration used to evaluate cellular viability).

**Figure 9:**
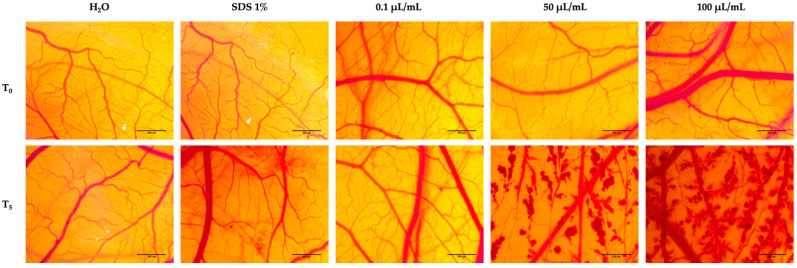
Stereomicroscopic images obtained before the application of the samples (T0) and five minutes after the application (T5) of CAMs inoculated with negative control—H_2_O (distilled water), positive control—SDS (sodium dodecylsulfate), and nicotine at 0.1, 50, and 100 μL/mL. The scale bars represent 500 µm.

Evaluations of the irritant potential were carried out based on the calculation of the irritation scores for both the control samples and for the test samples. Table 1 summarizes the results of the irritation scoring. According to the above results, the lowest irritation score was achieved with the negative control (distilled water), and the highest irritation score was achieved with the positive control (SDS 1%). A 0.1 μL/mL concentration of nicotine in the test samples produced a weak irritating response in the blood vessels, with the most prominent effect being slight intravascular coagulation and microhemorrhages. At the opposite pole was nicotine tested at a concentration of 50 and 100 μL/mL, for which we obtained irritation scores of 14.01 and 17.75, respectively, indicating a strong irritating effect. In addition, there was widespread generalized hemorrhaging along the entire surface of the membrane (Figure 9).

## 4. Discussion

As a matter of fact, the implications of smoking are well known, particularly in terms of cardiovascular and neoplastic diseases. It is also important to note that smoking has a toxic effect on the body, affecting a variety of cells in the body. Transdermal nicotine patches and smoking’s toxic effects may adversely affect keratinocytes. Nicotine is metabolized primarily by hepatocytes, which is why tobacco use leads to various toxic changes at this level [20]. Therefore, the current study was designed to assess the effects of nicotine on these cells at the cellular level. In addition, to gain a more comprehensive understanding of the toxic potential of nicotine, the HET-CAM method was applied to determine the potential for irritation of the chorioallantoic membrane of hen’s eggs.

In view of this, the first step in the study was to evaluate cell viability after stimulation for 24 h with five concentrations of nicotine (0.1, 10, 25, 50, and 100 μL/mL). By calculating the milligram equivalent based on nicotine density, the following concentrations were obtained: 0.10, 10.1, 25.2, 50.4, and 101 mg. The concentrations tested in this study were chosen primarily based on the nicotine content of electronic cigarettes. It has been demonstrated that there is a significant issue with the reporting of nicotine content. Additionally, it has also been established that the average nicotine content in electronic cigarettes varies between 6 and 36 mg/mL and can reach up to 60 mg/mL [21]. Moreover, the nicotine content of a cigarette was also considered, which varies depending on the type and size of the product, at a concentration of 6 to 28 mg/g of tobacco [22]. Nevertheless, the amount of nicotine absorbed after smoking a cigarette is approximately 1.1 to 1.5 mg nicotine per cigarette [23]. In view of the fact that a person can smoke on average 20 cigarettes per day, the amount of nicotine they can be exposed to in any given day ranges between 22 and 30 mg.

During the study, it was observed that nicotine can induce a marked reduction in cell viability, especially at the highest concentrations tested (50 and 100 μL/mL), resulting in an average viability of only 2% for all tested cell lines. Among the cell types studied, the only difference was that in human keratinocytes, the concentration of 25 μL/mL caused a reduction in viability to approximately 82 percent, whereas in hepatocytes and cardiomyocytes, the same concentration produced a marked reduction in viability of about 2 percent.

In one of the earliest studies on the effects of nicotine on human keratinocytes, it was found that nicotine was 50% absorbed in HaCaT cells three hours after exposure. Moreover, cotinine, one of the main metabolites of nicotine, has also been found in keratinocytes, indicating that nicotine is also metabolized in these cells [24]. A study by Pozuelos et al. examined the effect of nicotine on both keratinocyte cultures and a 3D model of human skin (EpiDermTM). The results showed that nicotine damages cellular organs, disrupts the homeostasis of reactive oxygen species, and causes oxidative damage to skin cells [25]. Likewise, Lee and colleagues examined the effects of nicotine on immortalized and malignant keratinocytes. Upon testing, the researchers found that nicotine had an anti-proliferative effect, depending on the exposure time and concentration. Furthermore, using 3D organotypic cultures, it has been demonstrated that high nicotine concentrations lead to impaired epithelial maturation, decreased surface keratinization, and decreased skin thickness [26]. It was observed that e-cigarette vapor had a pronounced cytotoxic effect on the HaCaT cell line, manifesting itself as breaks in DNA strands [27]. Additionally, Cervellati et al. determined the effect of cigarette smoke and e-cigarette vapor on the viability of HaCaT cells. They concluded that regular cigarettes, as well as e-cigarettes, have a significant cytotoxic effect on human keratinocytes [28]. Similarly, Shaikh et al. studied the effects of liquids found in electronic cigarettes on oral fibroblasts and oral keratinocytes. The authors reported that e-liquids had cytotoxic effects that depended on the concentration when cells were exposed for short to medium periods of time [29]. There are several possible mechanisms by which nicotine could cause toxicity in human keratinocytes, including the stimulation of nicotine-like acetylcholine receptors (nAChRs), thereby altering the skin’s barrier function [30]. There is evidence that nicotine exposure increases the levels of alpha7 nicotinic receptor subunits (α7-nAChRs) [31]. α7-nAChR plays a critical role in wound healing, regeneration of the epithelium, and inflammation suppression; however, when overactivated, it has the opposite effect [32].

Hepatocytes were significantly more likely to be influenced by nicotine stimulation as compared with keratinocytes, so cell viability decreased markedly, beginning at the concentration of 25 μL/mL. The cytotoxicity of flavoring chemicals used in e-cigarettes was also examined in a similar study using the HepG2 hepatocyte cell line. There is a constant drift of chemicals in an e-cigarette, which, as a result, has toxic effects on liver cells that cannot be directly correlated with nicotine content [33]. Likewise, Wieczorek et al. assessed the cytotoxic potential of nicotine HepG2 cells, as well as various electronic vaping products with different flavors. Nicotine was found to be more toxic than most of the products tested by the researchers [34]. In a murine study, the effect of nicotine on mouse hepatocytes was evaluated. Upon administration of nicotine for 24 days, nitric oxide levels, alanine aminotransferase, aspartate aminotransferase, and alkaline phosphatase levels increased, ultimately leading to detrimental effects on hepatocytes [35]. The toxic effect of nicotine on mouse hepatocytes was reported in a similar study on Swiss albino mice. It has been suggested that nicotine has hepatotoxic effects due to hepatocyte accumulation of iron, increased pro-inflammatory cytokine levels, and reduced glutathione peroxidase activity and glutathione levels [36].

Nicotine plays an important role in the development and progression of cardiovascular disease [37]. According to the present study, stimulation for a period of 24 h caused a marked reduction in the viability of cells of around 2%. A related study published by Huang et al. reported similar results to those of the present study, where nicotine significantly decreased the viability of H9C2 cells in a dose-dependent manner [38]. In the same manner, Wen-xia and colleagues determined that nicotine induces apoptosis in the H9C2 cell line, increasing the levels of caspase-3 cleavage, as well as Bcl2 and Bax expression [39]. Aside from these studies, Zhou et al. demonstrated that nicotine induces apoptosis in cardiomyocytes by increasing the level of oxidative stress and altering the expression of genes involved in apoptosis [17]. In a comparison conducted between the effects of cigarette smoke and electronic cigarette vapors on cardiomyocyte viability, it was found that e-vapors had a marked cytotoxic effect on the H9C2 cell line. However, the toxic effect was more pronounced in the case of regular cigarette smoke [40]. Additionally, further studies have demonstrated that cigarette smoke extract causes cardiomyocytes to undergo apoptosis and necrosis [41,42]. Smoke extract has been found to cause cytotoxicity primarily through increased oxidative stress and induced inflammation [43,44]. There has also been an increased emphasis on mitochondrial dysfunction and DNA impairment as potential mechanisms of cellular dysfunction [45]. It has also been suggested that the alteration of acetylcholine receptors may also be responsible for cardiotoxicity [46]. Therefore, when α7-nAChRs in cardiomyocytes, endothelia, and inflammatory cells are activated, endothelial dysfunction, inflammation, and myocardial changes occur [47]. The results of these studies are agreement with those obtained in the present investigation. Furthermore, by examining the cell morphologies and staining methods, it was confirmed that nicotine induced apoptotic changes in all three cell types examined.

Furthermore, a significant step in this study was the evaluation of the effect of nicotine on the blood vessels of the chorioallantoic membrane of hen’s eggs. According to our findings, the lowest concentration tested, 0.1 μL/mL, did not contribute significantly to changes in vascular architecture. In contrast, exposure to concentrations of 50 and 100 μL/mL induced widespread irritant effects, including hemorrhage, coagulation, and blood vessel lysis. In this case, the irritation score calculated for the two concentrations was analogous to that of the positive control, suggesting that nicotine is a highly irritant substance. To the best of our knowledge, the irritating effect on the chorioallantoic membrane has not yet been explored. The in ovo model, however, has been used to analyze the effects of nicotine on angiogenesis. Thus, in a study conducted by Mousa, the pro-angiogenetic role of nicotine was highlighted using the CAM method. This effect of stimulating the formation of new blood vessels is favorable during a tumor process [48]. As part of their study, Brown et al. evaluated the effects of nicotine on chorioallantoic membranes containing squamous cell adenocarcinomas that were previously treated with nicotine. According to the study results, nicotine led to increased expression of α7-nAChRs, which had a stimulating effect on tumor invasion and metastasis [49]. Alternatively, extracts from electronic cigarettes have been tested for their effect on angiogenesis and embryo development in the chorioallantoic membrane. Research has established that the extracts killed 70% of the embryos after a 5 day exposure period and, in addition, inhibited angiogenesis [50].

Even though extensive research has been conducted on the relationship between tobacco and nicotine use in health, this area has not been fully explored with consideration of the continuous growth of nicotine-containing products [51]. The tobacco industry’s involvement should also be mentioned, as it has challenged the findings in the field, making it even more difficult to understand the toxicological mechanisms behind nicotine [52].

## 5. Conclusions

Nicotine was shown to have cell-damaging effects on the three cell lines studied—keratinocytes, hepatocytes, and cardiomyocytes—depending on the test concentration. Furthermore, by visualizing the nucleus, it was observed that nicotine caused changes such as condensation and fragmentation of the nucleus, which are characteristic signs of cellular apoptosis. Moreover, the in ovo study, conducted on the vascular plexus of the chorioallantoic membrane, showed that nicotine had a strong irritating effect. In summary, these results support the toxicological profile of nicotine, which can be found in both regular cigarettes as well as electronic cigarettes, which are gaining ground steadily. Nevertheless, more research is necessary, both to shed light on the toxic mechanisms of nicotine as well as to determine how nicotine interacts with other compounds found in smoking products.

## Figures and Tables

**Table 1:** Irritation score values for positive controls (SDS 1%), negative controls (distilled water), and nicotine (0.1, 50, and 100 μL/mL).

	H_2_O	SDS 1%	0.1 μL/mL	50 μL/mL	100 μL/mL
IS	0.13	19.68	4.13	14.01	17.75
tH (s)	300	21	224	135	56
tL (s)	300	18	287	98	47
tC (s)	299	15	217	84	43

IS—irritation score; tH—hemorrhage time; tL—lysis time; tC—coagulation time; H_2_O—distilled water; SDS—sodium dodecylsulfate.

## Data Availability

The data presented in this study are available on request from the corresponding author.
